# Diet‐induced obesity decreases rate‐dependent depression in the Hoffmann’s reflex in adult mice

**DOI:** 10.14814/phy2.14271

**Published:** 2019-10-29

**Authors:** Gerard L. Nguyen, Shea Putnam, Mulatwa Haile, Zahra Raza, Martina Bremer, Katherine A. Wilkinson

**Affiliations:** ^1^ Department of Biological Sciences San José State University San Jose California; ^2^ Department of Mathematics and Statistics San José State University San Jose California

**Keywords:** Diet‐induced obesity, electrophysiology, Hoffmann’s reflex, rate dependent depression

## Abstract

Obesity is associated with balance and motor control deficits. We have recently shown that Group Ia muscle spindle afferents, the sensory arm of the muscle stretch reflex, are less responsive in mice fed a high‐fat diet. Here we test the hypothesis that reflex excitability to sensory information from Group Ia muscle spindle afferents is altered in a mouse model of diet‐induced obesity. We measured the anesthetized Hoffmann’s or H‐reflex, the electrical analog of the muscle stretch reflex. Adult mice of both sexes were fed a control diet (CD; 10% kcal from fat) or a high‐fat diet (HFD; 60% kcal from fat) for 5, 10, or 15 weeks. We used three quantitative measures of H‐reflex excitability: (1) H‐reflex latency; (2) the percentage of motor neurons recruited from electrical stimulation of Group Ia muscle spindle afferents (H_max_/M_max_); and (3) rate‐dependent depression (RDD), the decrease in H‐reflex amplitude to high frequency stimulation (20 stimuli at 5 Hz). A HFD did not significantly alter H latency (*P* = 0.16) or H_max_/M_max_ ratios (*P* = 0.06), but RDD was significantly lower in HFD compared to CD groups (*P* < 0.001). Interestingly, HFD males exhibited decreased RDD compared to controls only after 5 and 10 weeks of feeding, but females showed progressive decreases in RDD that were only significant at 10 and 15 weeks on the HFD. These results suggest that high‐fat feeding increases H‐reflex excitability. Future studies are needed to determine whether these changes alter muscle stretch reflex strength and/or balance and to determine the underlying mechanism(s).

## Introduction

Obesity is a metabolic disorder characterized by excess adiposity and chronic low‐grade systemic inflammation that often occurs with cardiovascular disease, arthritis, and type 2 diabetes (Kopelman 2007). Obesity is also linked to an increased risk of falling (Corbeil et al. 2001; Fjeldstad et al. 2008; Madigan et al. 2014) and an increased risk of fall‐related injuries (Matter et al. 2007; Himes and Reynolds 2012). Individuals with obesity exhibit balance deficits due to increased sway during standing (Greve et al. 2007) and altered gait (Lai et al. 2008; Ko et al. 2010), both of which are risk factors for falling (Ganz et al. 2007). Mice fed a high‐fat diet exhibit similar deficits in gait (Takase et al. 2016) and balance (Griffin et al. 2010; Lee et al. 2015) as humans. The increased sway during standing seen in people with obesity is at least partially due to increased body weight (McGraw et al. 2000; Hue et al. 2007; Teasdale et al. 2007) and a shifted center of mass (Corbeil et al. (2001)). However, increased body weight and changes in center of mass cannot be the only contributors to the observed balance deficits and there is increasing evidence of somatosensory dysfunction during obesity (Handrigan et al. 2012; Mignardot et al. 2013). Individuals with obesity have more difficulty stabilizing in the absence of visual cues (McGraw et al. 2000; Cruz‐Gómez et al. 2011) and foot plantar mechanoreceptors in humans (Rocha et al. 2014; Wu and Madigan 2014) and muscle spindle afferents in mice (Elahi et al. 2018) exhibit decreased responsiveness during obesity.

Whether central integration of somatosensory information is also compromised during obesity is not well understood and could contribute to balance instability. The muscle stretch reflex is important for balance control and error correction during ongoing movement and has a strong influence on postural sway (Dietz 2002; Gandevia et al. 2012). The Group Ia muscle spindle afferents sense changes in muscle length and synapse directly onto motor neurons, causing a reflex contraction. Changes in muscle stretch reflex strength could contribute to balance instability (Gandevia et al. 2012). We previously reported a decreased response to stretch by muscle spindle afferents in mice (Elahi et al. 2018), which could lead to decreased reflex strength if not compensated for with increased reflex excitability.

We tested the hypothesis that high‐fat feeding would alter reflex excitability to Group Ia muscle spindle afferent activity using the Hoffmann’s reflex (H‐reflex), the electrical analog of the muscle stretch reflex. Mice of both sexes were fed a control or high‐fat diet for 5, 10, or 15 weeks. We used three measures of H‐reflex excitability: H‐reflex latency, percentage of available motor neurons (M_max_) activated by electrical stimulation of Group Ia afferents (H_max_/M_max_), and rate‐dependent depression of the H wave amplitude at high frequency stimulation (RDD; 20 stimuli at 5 Hz). In humans, body mass index was not correlated with H‐reflex latency, but other measures of H‐reflex excitability were not reported (Buschbacher 1998). Here we report increased H‐reflex excitability as measured by RDD in mice fed a HFD, as well as sex differences in the time course of the excitability changes observed.

## Methods

### Animals and diets

All procedures were approved by the San José State University Institutional Animal Care and Use Committee (IACUC). Four‐week‐old C57BL/6 mice of both sexes were purchased from Simonsen Laboratories (Gilroy, CA). Upon arrival, the mice were weighed, ear‐notched for identification, and acclimatized for one week on a control diet (CD; 10% kcal from fat; D12450J, Research Diets, New Brunswick, NJ) and given water ad libitum. All mice were housed in cages of five to six individuals under a 12:12‐h light:dark cycle. After the acclimatization period, half of the cages were randomly switched to a high‐fat diet (HFD; 60% kcal from fat; D12492; Research Diets) while the other half remained on the CD for 5, 10, or 15 weeks. The mice were weighed weekly for the duration of the experiments.

### Hoffmann’s reflex testing

A subcutaneous injection of a ketamine (125 mg/kg) and xylazine (12.5 mg/kg) was used to anesthetize the mice. Ketamine and xylazine were chosen because they do not reduce reflex excitability, unlike other anesthetics that enhance GABA‐mediated presynaptic inhibition (Ho and Waite 2002). Anesthetic depth during the experiment was tested using a toe pinch every 30 min. Body temperature was monitored using a rectal thermometer (BAT‐12, Physitemp, Clifton, NJ) and maintained by running warm water through a surgical platform (Part #809B, Aurora Scientific, Aurora, Ontario, Canada).

We measured the Hoffmann’s reflex (H‐reflex), the electrical analog of the muscle stretch reflex. To do this, we made an incision on the thigh to expose the sciatic nerve and placed bipolar tungsten stimulating electrodes (A‐M Systems, Sequim, WA) on the nerve. Hip, knee, and ankle joints were stabilized at 90° angles using pieces of Sylgard pinned into place. We placed recording electrodes in the fourth dorsal interosseous muscle of the foot. This muscle was chosen because it yielded a clear separation in time between the first muscle contraction wave caused by direct stimulation of alpha motor neurons (M wave) and the reflex muscle contraction caused by Group Ia electrical stimulation (H wave). Muscle EMGs were recorded using an extracellular bio‐amplifier (DAM50; World Precision Instruments, Sarasota, FL) and digitized for analysis (PowerLab and LabChart software; AD Instruments, Colorado Springs, CO).

We first determined the threshold voltage of the H wave (T) using a 0.2‐msec square pulse stimuli (Grass Instruments S88, Quincy, MA). We stimulated the nerve 20 times at 0.1 Hz frequency at increasing voltages calculated as factors of T (T, 1.3T, 1.5T, 2T, 3T, 5T, 6T, 7T, and 8T). H wave threshold was then re‐verified and the stimulus trains (20 0.2 msec square pulses) repeated at 5 Hz. After experimentation, mice were euthanized by cervical dislocation.

To determine maximum M and H peak‐to‐peak amplitudes, we averaged all 20 traces at each stimulus threshold at 0.1 Hz. We determined the maximum percentage of motor neurons that could be excited by Group Ia stimulation by dividing the maximum H amplitude by the maximum M amplitude (H_max_/M_max_) (Knikou 2008). M and H latencies were measured as the time to onset of each wave at the stimulus thresholds that yielded the maximum M and H amplitudes, respectively. We calculated percent rate‐dependent depression (RDD) observed following a train of 20 stimuli given at 5 Hz at 1.5 T stimulus strength. Higher frequency stimuli are known to decrease the amplitude of the H reflex to a stable lower amplitude after the first 3–5 stimuli, so we expressed the average H amplitude of the 6th‐20th stimulations as a percentage of the initial H amplitude in the stimulus train. In four cases we were not able to record from the same animal at both the 0.1 Hz and the 5 Hz stimulation trains (1 M CD 5 weeks animal is missing RDD data; 1 F CD 5 weeks, 1 M CD 10 weeks, and 1 M HFD 10 weeks animals are missing M and H latency and H_max_/M_max_ data).

### Measurement of serum concentrations of leptin and TNF‐*α*

After the electrophysiological experiments, 250–300 *µ*L of blood was collected from each mouse via retro‐orbital bleeding into a 1.5 mL microcentrifuge tube. The blood was allowed to clot at room temperature for 20–30 min before centrifugation at 3000 rcf for 15 min. 60 *µ*L of serum was transferred to a separate microcentrifuge tube and stored at −80°C. A custom Quantibody ELISA kit (RayBiotech, Norcross, GA) was used to determine serum concentrations of leptin and tumor necrosis factor alpha (TNF‐*α*). The slides were blocked for 2 h before incubation overnight (12 h) at 4°C. Samples from males fed for 10 weeks were assayed several months before the rest of the samples to ensure the assay worked. Because control levels of TNF‐*α* (generalized least squares, *P* = 0.96) and leptin (*P* = 0.17) were similar in both experiments, we presented results from all groups together.

### Statistics

To account for unequal sample variances among three or more sample groups, generalized least squares (GLS) tests were performed using R Statistical Software (v. 3.5.1) to test for the overall effects of diet, sex, and time on the diet. Similarly, for two‐sample group tests with unequal variances, we performed Welch’s *t*‐tests using IBM SPSS Statistics (v. 24) to test for significant diet effects in each sex at all three time points. The *t*‐test *P*‐

values comparing CD versus HFD groups are reported in text in order of increasing time on the diet, with male groups at each time point listed first. For all tests, *P* < 0.05 was considered significant. In the figures, horizontal black bars represent the mean of each group and error bars represent the standard error of the means.

## Results

### Body weights increased after high‐fat feeding

**Figure 1:**
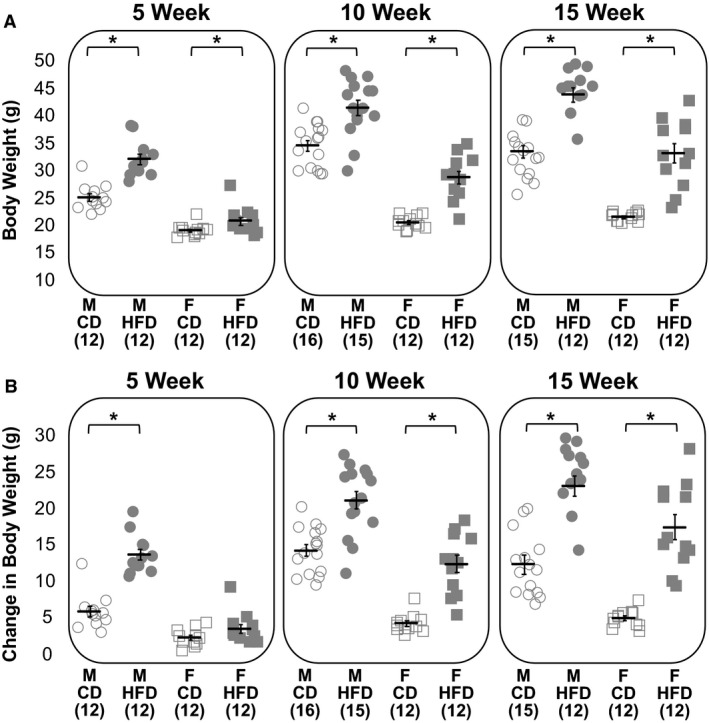
Body weight and weight gains were greater following high‐fat feeding. Final body weights (A) and changes in body weight from day of arrival to day of experiment (B) for males (circles) and females (squares) on a CD (open) and a HFD (filled). The numbers in parentheses below the *X*‐axis indicate group sample size. Horizontal black bars represent group means. Error bars represent the standard error of the mean. Asterisks denote statistical significance (*P* < 0.05) according to Welch’s *t*‐tests against relevant controls.

Mice of both sexes fed a HFD weighed significantly more and gained significantly more weight than mice fed a CD (GLS, *P* < 0.001, *P* < 0.001, Fig. 1). At all time points, mice fed a HFD weighed significantly more than the control group of the same sex (Welch’s *t*‐test, M 5 weeks *P* < 0.001, F 5 weeks *P* = 0.04, M 10 weeks *P* = 0.001, F 10 weeks *P* < 0.001, M 15 weeks *P* < 0.001, F 15 weeks *P* < 0.001; Fig. 1A). Similarly, all HFD animals gained significantly more weight than their control group, with the exception of females at 5 weeks (Welch’s *t*‐test, M 5 weeks *P* < 0.001, F 5 weeks *P* = 0.11, M 10 weeks *P* < 0.001, F 10 weeks *P* = 0.01, M 15 weeks *P* < 0.001, F 15 weeks *P* < 0.001, Fig. 1B). On average, males had heavier body weights and exhibited greater weight gains than females (GLS, *P* < 0.001, *P* < 0.001).

### Serum leptin but not TNF‐*α* levels increased after high‐fat feeding

**Figure 2:**
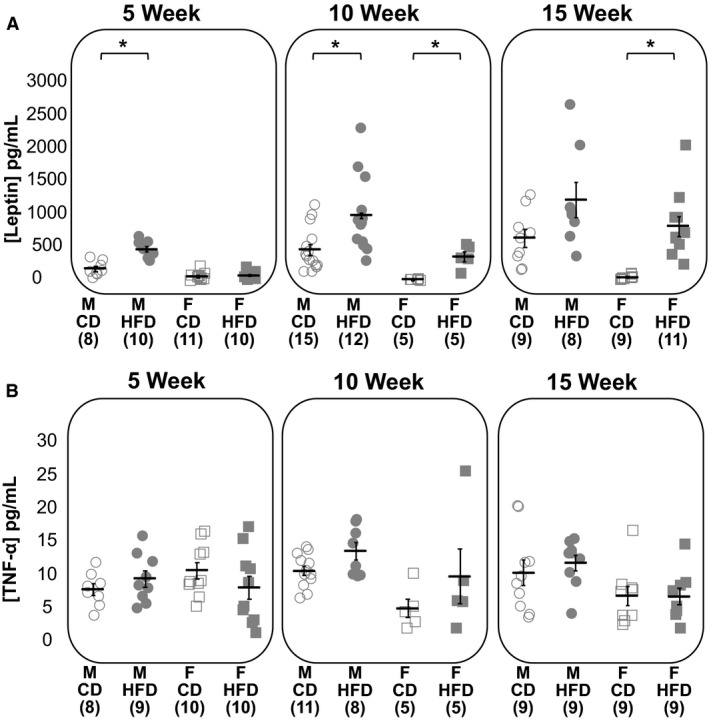
Serum leptin but not TNF‐*α* levels increased following high‐fat feeding. Serum levels of leptin (A) and TNF‐*α* (B) for males (circles) and females (squares) on a CD (open) and HFD (filled) measured with an ELISA. The numbers in parentheses on the *X* axis indicate each group’s sample size. Horizontal black bars represent the means. Error bars represent the standard error of the mean. Asterisks denote statistical significance (*P* < 0.05) according to Welch’s *t*‐tests.

On average, HFD groups had significantly greater serum leptin levels than CD groups (GLS, *P* < 0.001; Fig. 2A). Leptin levels were significantly greater in HFD groups than CD groups at all time points except for in 5 week females and 15 weeks males (Welch’s *t*‐test, M 5 weeks *P* < 0.001, F 5 weeks *P* = 0.56, M 10 weeks *P* = 0.01, F 10 weeks *P* = 0.006, M 15 weeks *P* = 0.07, F 15 weeks *P* = 0.001; Fig. 2A). Leptin also increased progressively in both sexes the longer the animal was on the HFD (GLS, 5 weeks vs. 10 weeks *P* = 0.003, 10 weeks vs. 15 weeks *P* = 0.02; Fig. 2A). Averaged across all time points, males had significantly higher serum leptin levels than females (GLS, *P* < 0.001).

High‐fat feeding had no significant overall effect on serum TNF‐*α* concentrations (GLS, *P* = 0.20; Fig. 2B). Compared to matched controls, high‐fat feeding did not significantly alter serum TNF‐*α* concentrations at any time point (Welch’s t‐test, M 5 weeks *P* = 0.31, F 5 weeks *P* = 0.25, M 10 weeks *P* = 0.05, F 10 weeks *P* = 0.31, M 15 weeks *P* = 0.52, M 15 weeks *P* = 0.82). On average, males had greater serum TNF‐*α* levels than females (GLS, *P* = 0.01; Fig. 2B).

### M and H latencies were largely unchanged by high‐fat feeding

**Figure 3:**
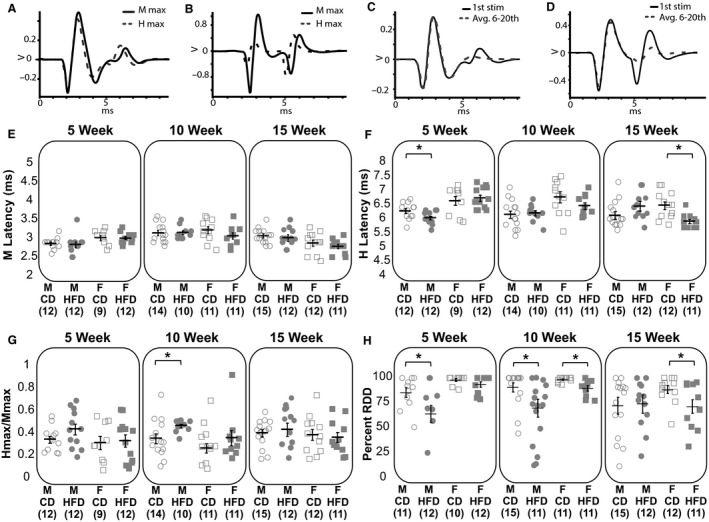
H‐Reflex excitability increased following high‐fat feeding. Representative traces showing the stimulations that produced the M_max_ (solid line) and H_max_ (dashed line) waves in a CD animal (A) and a HFD animal (B). Representative result showing complete RDD from a CD animal (C), and incomplete RDD from a HFD animal (D). Individual data for M latencies (E), H latencies (F), H_max_/M_max_ ratio (G), and percent RDD (H) are shown for males (circles) and females (squares) on a CD (open) or HFD (filled). Horizontal black bars represent the mean. Error bars represent the standard error of the mean. Asterisks denote statistical significance (*P* < 0.05) according to Welch’s *t*‐tests.

High‐fat feeding did not affect the latency of the M wave (GLS, *P* = 0.28; Fig. 3E) and there were no sex differences in M latency (GLS, *P* = 0.46). Similarly, no group differences at any time point were observed (Welch’s *t*‐test, M 5 weeks *P* = 0.70, F 5 weeks *P* = 0.96, M 10 weeks *P* = 0.92, F 10 weeks *P* = 0.23, M 15 weeks *P* = 0.60, F 15 weeks *P* = 0.38). High‐fat feeding did not affect the latency of the H wave overall (GLS, *P* = 0.36; Fig. 3F), but did significantly decrease the H latencies for 5‐week males and 15‐week females (Welch’s *t*‐test, M 5 weeks *P* = 0.03, F 5 weeks *P* = 0.61, M 10 weeks *P* = 0.77, F 10 weeks *P* = 0.18, M 15 weeks *P* = 0.14, F 15 weeks *P* = 0.002; Fig. 3F). On average, males had shorter H latencies than females (GLS, *P* = 0.001).

### H_max_/M_max_ unchanged following high‐fat feeding

On average, HFD groups did not have significantly higher H_max_/M_max_ ratios than CD groups, although it was approaching significance (GLS, *P* = 0.06). Only males fed a HFD for 10 weeks showed a significantly higher H_max_/M_max_ than their controls (Welch’s *t*‐test, M 5 weeks *P* = 0.13, F 5 weeks *P* = 0.83, M 10 weeks *P* = 0.03, F 10 weeks *P* = 0.26, M 15 weeks *P* = 0.23, F 15 weeks *P* = 0.72; Fig. 3G). On average, males had a greater H_max_/M_max_ ratio compared to females (GLS, *P* = 0.01; Fig. 3G). There was no significant difference in the stimulation strength that led to the maximal H wave amplitude based on diet (3 factor ANOVA; *P* = 0.10), sex (*P* = 0.92), or weeks on the diet (*P* = 0.20).

### Rate‐dependent depression decreased after high‐fat feeding

On average, HFD groups had significantly less RDD than CD groups (GLS, *P* < 0.001; Fig. 3H). Mice fed a HFD had significantly less RDD than the control group of the same sex at all time‐points except for 5‐week females and 15‐week males (Welch’s *t*‐test, M 5 weeks *P* = 0.04, F 5 weeks *P* = 0.13, M 10 weeks *P* = 0.03, F 10 weeks *P* = 0.01, M 15 weeks *P* = 0.99, F 15 weeks *P* = 0.04). On average, males had significantly less RDD than females (GLS, *P* < 0.001; Fig. 3H).

## Discussion

### Rate‐dependent depression decreased in mice of both sexes fed a HFD

In this study, we tested the hypothesis that high‐fat feeding would alter spinal cord processing of sensory information from Group Ia muscle spindle afferents by measuring the H‐reflex in male and female mice fed a HFD for 5, 10, or 15 weeks. The HFD groups were significantly heavier than matched controls at all time points, although females at 5 weeks did not gain significantly more weight than their matched control group. We found that one measure of H‐reflex excitability to Group Ia sensory input, RDD, was altered in mice fed a HFD. The changes observed had a different time course of onset in males and females. Male mice showed significantly decreased RDD at 5 and 10 weeks but not at 15 weeks on the HFD. Female mice, however, only exhibited decreased RDD at 10 and 15 weeks on the diet. The percentage of motor neurons recruited by electrical stimulation (H_max_/M_max_) was trending higher in HFD animals, but only 10‐week HFD males showing significantly larger H_max_/M_max_ ratios. The reliability of the H_max_/M_max_ measure can be influenced by experimental factors, including joint position (Merlet et al. 2018) and anesthetic state (Ho and Waite 2002). These factors were controlled as much as possible, but day to day variation could have existed and limited our ability to see larger group differences. Also consistent with increased H‐reflex excitability, we found decreased H latencies in 5‐week HFD males and 15‐week HFD females, although there was no overall effect of diet. Since latency is highly dependent on electrode placement, we tried to use consistent electrode placements. However, daily variation likely still occurred and the latency results should be interpreted with caution. Overall, the changes observed suggest modest increases in H‐Reflex excitability following high‐fat feeding.

Overall, males exhibited increased H reflex excitability at the earlier time points, but not at 15 weeks. Females showed progressive increases in reflex excitability with the largest changes observed following 15 weeks on the diet. Interestingly, this pattern matched that seen in serum leptin levels and to some extent body weight changes, potentially suggesting metabolic changes as causal. Significantly higher leptin levels were seen in males on the HFD only at 5 and 10 weeks and in female mice only at 10 and 15 weeks. Females showed relatively small differences in body weight at 5 weeks, with increasing group differences in later time points. Female control mice at all three time points had similar leptin levels. Males, however, showed the clearest distinction in group weights at the early time points. Similarly, male control leptin levels increased in the later time points, suggestive of metabolic changes at 15 weeks in control males that might have masked the effect of high‐fat feeding. Future studies could try to prevent these metabolic changes in control mice with access to an exercise wheel or other interventions.

In contrast, we saw no significant changes in serum TNF‐*α* levels in HFD mice, even though high‐fat feeding is known to cause chronic inflammation (Gregor and Hotamisligil 2011). However, serum TNF‐*α* levels were very low and close to the limit of detection for our ELISA. Future testing could use more sensitive methods of measuring inflammatory state, such as inflammatory markers directly from the fat tissue. Due to these limitations, we cannot conclude whether changes in inflammatory state correlate with the changes in H‐reflex excitability that we have observed.

### Physiological significance

We observed an approximately 10% decrease in RDD in HFD mice, which is statistically significant but smaller in magnitude than the 30‐50% decreases in RDD seen in diabetic rat models of both type 1 and type 2 diabetes (Jolivalt et al. 2008; Lee‐Kubli and Calcutt 2014; Marshall et al. 2017), post‐stroke rats (Toda et al. 2014), and spinal cord injured rats (Thompson et al. 1992; Boulenguez et al. 2010), which are conditions also associated with postural instability (Simoneau et al. 1994; Hyndman et al. 2002; Brotherton et al. 2007). Interestingly, the obese Zucker Diabetic Fatty rat model of type 2 diabetes showed an ~35% decrease in RDD after developing symptoms of tactile allodynia, but not at earlier time points even though they were hyperglycemic (Marshall et al. 2017). High‐fat feeding in C57BL/6 mice leads to the progressive development of pre‐diabetic symptoms including hyperinsulinemia, impaired glucose tolerance, and neuropathy. The time course of the development of prediabetes varies based on sex and the time the diet was started (O'Brien et al. 2014; Kleinert et al. 2018). A limitation of our study was that we did not carefully measure metabolic indices over the 15 weeks of feeding. Our mice at most time points were likely pre‐diabetic given their leptin levels, but are unlikely to have developed type 2 diabetes (Kleinert et al. 2018), which could explain the less severe phenotype we observed as compared to the Zucker Diabetic Fatty rats. Similarly, male mice are known to experience metabolic changes earlier than female mice on a high‐fat diet (Pettersson et al. 2012; O'Brien et al. 2014), which is consistent with the earlier appearance of decreased RDD in male mice. Future studies should more carefully determine the relationship between metabolic changes and changes in RDD.

We would predict hyperexcitability of the stretch reflex based on the increased H‐reflex excitability we observed. However, previous work in our laboratory demonstrated decreased muscle spindle afferent response to muscle stretch in HFD mice (Elahi et al. 2018), and this decreased sensory input would suggest decreased stretch reflex strength. It is possible that the decreased input from muscle spindle afferents could be compensated for by the increased H reflex excitability we observed, or that one effect is stronger and ultimately alters stretch reflex strength. Very few studies have directly tested stretch reflex strength during obesity and have only reported reflex latency results. Half‐relaxation time of the Achilles tendon reflex was found to be unchanged in people with obesity (Burt and Stunkard 1964; Glennon and Reinfrank 1966; Reinfrank et al. 1967), similar to the unchanged H latency observed in humans (Buschbacher 1998) and in most of our sample groups. Future studies will be needed to determine whether stretch reflex strength changes during obesity in our mouse model or in humans. If stretch reflex strength is altered, it could be a contributing factor to the balance instability and increased risk of falling seen during obesity (Corbeil et al. 2001; Fjeldstad et al. 2008; Madigan et al. 2014).

### Potential mechanisms for increased h‐reflex excitability

Changes in H‐reflex excitability could be caused by changes in synaptic efficiency, presynaptic inhibition, and/or motor neuron excitability (Misiaszek 2003). High‐fat feeding may lead to sprouting of additional afferent terminals onto motor neurons, as occurs in spinal cord injured rats, which could contribute to decreased RDD and hyperreflexia (Tan et al. 2012). In spasticity after stroke and spinal cord injury, decreased RDD has been attributed to decreased expression of the potassium‐chloride cotransporter KCC2 in motor neurons (Boulenguez et al. 2010; Toda et al. 2014). This leads to higher intracellular chloride levels and reduces or reverses the inhibitory effect of GABA neurotransmission (Boulenguez et al. 2010). In the STZ‐injected rat model of type 1 diabetes, there is decreased expression of KCC2 in the dorsal horn of the spinal cord but not the ventral horn motor neurons. In diabetes, this reduction in dorsal horn KCC2 levels may alter RDD through yet uncharacterized dorsal horn projections on the primary afferents or oligosynaptic excitatory connections onto motor neurons. Future studies are needed to determine whether our mouse model of diet‐induced obesity results in the downregulation of KCC2 in the dorsal or ventral horns, sprouting of Group Ia afferent terminals onto motor neurons, and/or other mechanisms that could alter H‐reflex excitability.

### Sex differences in the H‐reflex

We also found a differing time course for the development of H‐reflex hyperexcitability and increased serum leptin levels between males and females, which is suggestive of sex differences in the progression of obesity‐related changes. In males, changes were present after 5 and 10 weeks on a HFD but not after 15 weeks. Conversely, these changes were not seen in females until 10 weeks on a HFD. Interestingly, these same time‐dependent sex differences were observed in serum leptin levels, which may suggest a causal role for metabolic changes in the development of H reflex hyperexcitability as described above. Similarly, unlike the stable control leptin levels in females, male control leptin levels increased at the later time points, suggesting that our control animals may have also have been undergoing metabolic changes that may explain the lack of difference between the control and HFD group at 15 weeks in male mice. The initial protection for females may be at least partially explained by the actions of estrogen, which has been suggested to be protective against weight gain and metabolic alterations with a HFD (Lovejoy and Sainsbury 2009; Hwang et al. 2010). Determining the reason for the sex differences we have observed may suggest therapeutic targets for normalizing H reflex excitability and/or sex specific treatment strategies.

Overall, we found greater H‐reflex excitability in control males than in females. To the best of our knowledge, this is a novel observation as most studies measuring the H‐reflex have only used a single sex or have not reported any differences between sexes. Similarly, few studies have reported muscle stretch reflex sex differences. However, a study of human jaw muscle stretch reflexes showed greater reflex amplitude in females than in males (Peddireddy et al. 2006), while a study of back reflexes showed larger reflex amplitudes in males (Larivière et al. 2010). Further studies are needed to better understand whether there are consistent sex differences in H‐reflex and/or stretch reflex amplitudes and the physiological relevance of those differences.

## Conclusion

In summary, RDD is decreased following high‐fat feeding in mice of both sexes. Further studies are needed to determine whether this leads to changes in muscle stretch reflex strength and contributes to the balance deficits observed during obesity. Sex differences were observed in the time course of these H‐reflex excitability changes, which suggests that future studies should include subjects from both sexes. Determining whether stretch reflex alterations contribute to balance deficits can inform therapies to reduce and prevent falls during obesity.

## Conflict of Interest

The authors have no conflicts of interest to declare.
